# *PFKFB2* Promoter Hypomethylation as Recurrence Predictive Marker in Well-Differentiated Thyroid Carcinomas

**DOI:** 10.3390/ijms20061334

**Published:** 2019-03-16

**Authors:** Mateus Camargo Barros-Filho, Larissa Barreto Menezes de Lima, Mariana Bisarro dos Reis, Julia Bette Homem de Mello, Caroline Moraes Beltrami, Clóvis Antonio Lopes Pinto, Luiz Paulo Kowalski, Silvia Regina Rogatto

**Affiliations:** 1 International Research Center-CIPE-A.C.Camargo Cancer Center, São Paulo 01508-010, Brazil; mfilho@accamargo.org.br (M.C.B.-F.); larissamenezes@outlook.pt (L.B.M.d.L.); marianabisarro@yahoo.com.br (M.B.d.R.); juliahmello@gmail.com (J.B.H.d.M.); cahbeltrami@gmail.com (C.M.B.); 2 Department of Pathology, A.C.Camargo Cancer Center, São Paulo 01509-900, Brazil; calopes@accamargo.org.br; 3 Department of Head and Neck Surgery and Otorhinolaryngology, A.C. Camargo Cancer Center, São Paulo 01509-900, Brazil; lp_kowalski@uol.com.br; 4 Department of Clinical Genetics, Vejle Hospital, Institute of Regional Health Research, University of Southern Denmark, 7100 Vejle, Denmark

**Keywords:** DNA methylation, *PFKFB2*, well-differentiated thyroid carcinoma, prognosis, *TERT* promoter mutation, *BRAF* mutation

## Abstract

Despite the low mortality rates, well-differentiated thyroid carcinomas (WDTC) frequently relapse. *BRAF* and *TERT* mutations have been extensively related to prognosis in thyroid cancer. In this study, the methylation levels of selected CpGs (5-cytosine-phosphate-guanine-3) comprising a classifier, previously reported by our group, were assessed in combination with *BRAF* and *TERT* mutations. We evaluated 121 WDTC, three poorly-differentiated/anaplastic thyroid carcinomas (PDTC/ATC), 22 benign thyroid lesions (BTL), and 13 non-neoplastic thyroid (NT) tissues. *BRAF* (V600E) and *TERT* promoter (C228T and C250T) mutations were tested by pyrosequencing and Sanger sequencing, respectively. Three CpGs mapped in *PFKFB2*, *ATP6V0C*, and *CXXC5* were evaluated by bisulfite pyrosequencing. *ATP6V0C* hypermethylation and *PFKFB2* hypomethylation were detected in poor-prognosis (PDTC/ATC and relapsed WDTC) compared with good-prognosis (no relapsed WDTC) and non-malignant cases (NT/BTL). *CXXC5* was hypomethylated in both poor and good-prognosis cases. Shorter disease-free survival was observed in WDTC patients presenting lower *PFKFB2* methylation levels (*p* = 0.004). No association was observed on comparing *BRAF* (60.7%) and *TERT* (3.4%) mutations and prognosis. Lower *PFKFB2* methylation levels was an independent factor of high relapse risk (Hazard Ratio = 3.2; CI_95%_ = 1.1–9.5). *PFKFB2* promoter methylation analysis has potential applicability to better stratify WDTC patients according to the recurrence risk, independently of *BRAF* and *TERT* mutations.

## 1. Introduction

Papillary (PTC) and follicular (FTC) thyroid carcinomas are the most prevalent thyroid tumors, designated as well-differentiated thyroid carcinomas (WDTC) [1]. Despite the high rates of recurrence, the majority of patients presents good prognosis, due to the low propensity for metastasis and excellent response to the conventional therapies [1]. Currently, clinical-pathological parameters used to predict the risk of relapse or mortality of WDTC patients are insufficient to accurately predict the disease progression [2,3,4]. The use of molecular approaches has the potential to reveal markers that can be used for risk stratification of these patients.

Papillary thyroid cancer harboring *BRAF* mutation is frequently described in patients with worse prognosis [5,6,7], mainly in older individuals [8]. However, the presence of this mutation is not enough to predict the risk of recurrence or death [9,10]. Recently, promoter mutations in the telomerase reverse transcriptase encoding gene (*TERT*) have been consistently associated with more aggressive thyroid carcinomas [11], especially in the presence of *BRAF* mutation [12,13]. *TERT* promoter mutation has been reported in approximately 9% of PTC and in higher frequency in poorly-differentiated thyroid carcinomas (PDTC) (40%) and anaplastic thyroid carcinomas (ATC) (>70%) [1]. Curiously, *TERT* promoter mutation is highly prevalent in advanced PTC (61% of the cases) and FTC (71%) [14]. This gene encodes the catalytic subunit of telomerase, a ribonucleoprotein complex that maintains the telomere length, playing an important role in the tumorigenesis and cellular immortality [15]. Two mutations in hot spots, C228T and C250T, mapped in the promoter region of this gene have been described in thyroid cancer [16].

In addition, the DNA methylation patterns have also been described with potential applicability in the prognosis of thyroid carcinomas [17,18,19,20,21]. At least two candidate markers of recurrence, *RUNX3* hypermethylation [19] and *TSHR* hypomethylation [17], were reported in PTC patients. However, investigations of epigenetic prognostic markers in WDTC are poorly explored in the literature. Even though the recurrence may occur many years after the initial treatment [22,23], the thyroid cancer patients enrolled in these studies frequently have a follow-up period shorter than five years.

In a previous study of our group, a genome-wide DNA methylation profiling (Illumina 450k platform) was performed in 141 thyroid samples, including WDTC, PDTC/ATC, benign thyroid lesions (BTL), and non-neoplastic thyroid (NT) tissues [20]. A prognostic signature was designed based on 21 CpGs (5-cytosine-phosphate-guanine-3), achieving 63% of sensitivity and 92% of specificity to discriminate recurrent from non-recurrent WDTC patients. A “high-risk” prediction was considered as an independent marker of worse prognosis in the multivariate analysis, which was reproducible using the PTC cohort from The Cancer Genome Atlas (TCGA) database [20].

Herein, *TERT* promoter and *BRAF* mutations were genotyped, and three differentially methylated CpGs, included in the previous reported prognostic signature, were assessed by bisulfite pyrosequencing. The molecular data were compared with the WDTC patient’s outcome, which was followed-up during 10.6 years in the median.

## 2. Results

**Figure 1:**
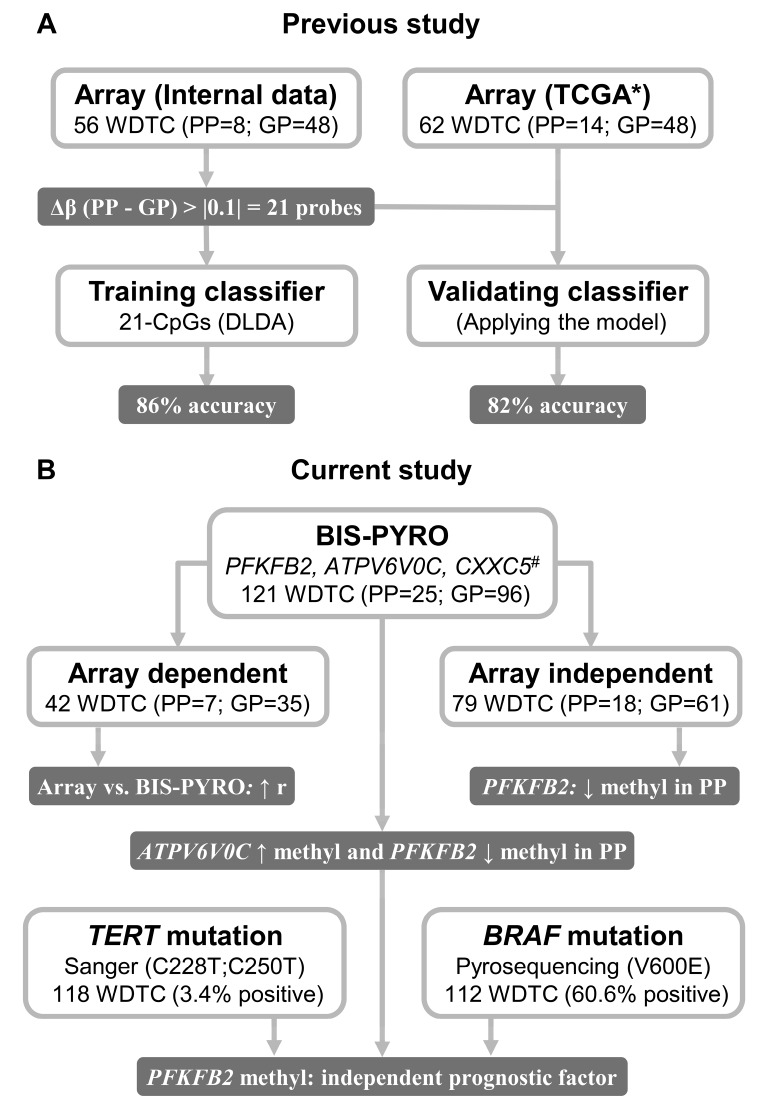
Workflow with the summarized study design and main results. (**A**) In the previous study [20], we developed a 21-CpG (5-cytosine-phosphate-guanine-3) prognostic classifier, presenting a high performance in discriminating WDTC-PP from WDTC-GP. (**B**) In the current study, we evaluated three CpGs from the previous classifier and tested the prognostic potential in array-dependent and independent WDTC samples. This analysis revealed *PFKFB2* hypomethylation as an independent poor prognostic factor. WDTC: well-differentiated thyroid carcinomas; PP: poor prognosis; GP: good prognosis; Δβ: delta of the mean methylation values (beta) from PP and GP cases; DLDA: Diagonal Linear Discriminant Analysis; BIS-PYRO: bisulfite pyrosequencing; ↑: high; ↓: low; * PTC patients from The Cancer Genome Atlas (TCGA) cohort elected based on the inclusion criteria [20]; ^#^ selection of three probes confirmed by TCGA (Δβ > |0.1|) presenting high AUC in the internal microarray data; r: correlation coefficient; methyl: methylation.

The study design and main results are summarized in Figure 1.

### 2.1. High Agreement between Global DNA Methylation and Bisulfite Pyrosequencing Results

High-quality the sequencing was obtained in 152, 146, and 159 samples for the CpGs mapped in the promoters of *PFKFB2*, *ATPV6V0C*, and *CXXC5* genes, respectively. Methylation microarray and pyrosequencing were both performed in 42 samples, showing high positive correlation (*PFKFB2 r* = 0.768; *ATPV6V0C r* = 0.762; *CXXC5 r* = 0.881) (Figure S1).

### 2.2. PFKFB2 Hypomethylation in Thyroid Carcinomas in Poor Prognosis Patients

**Figure 2:**
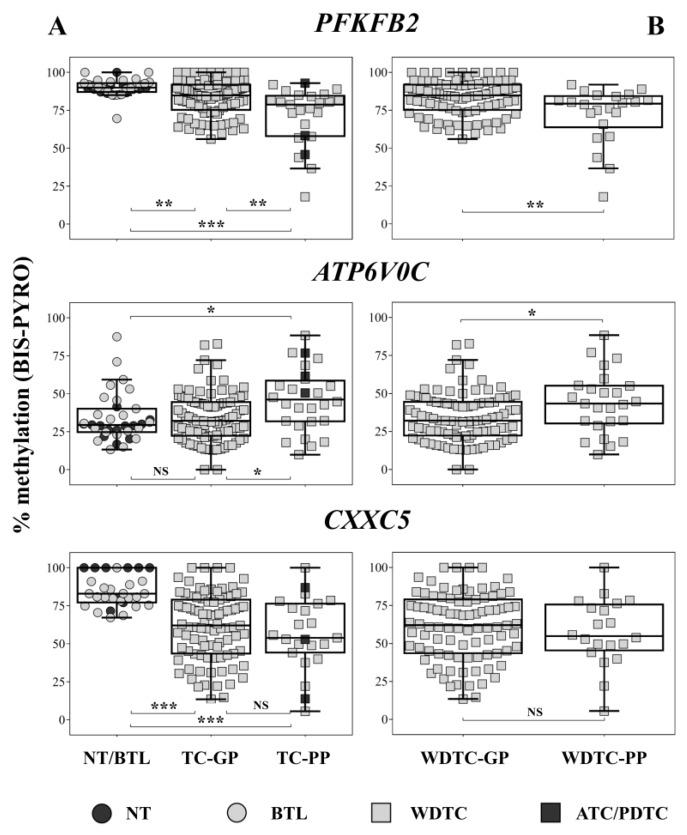
Bisulfite pyrosequencing quantification of CpG methylation in *PFKFB2*, *CXXC5,* and *ATPV6V0*. (**A**) Kruskal-Wallis and Dunn’s post-hoc tests were applied to compare non-neoplastic thyroid samples (NT/BTL) and carcinomas from patients with good (TC-GP) and poor prognosis (TC-PP). (**B**) Comparison between poor prognosis (WDTC-PP) and good prognosis (WDTC-GP) well-differentiated thyroid carcinomas using Mann Whitney test. BIS-PYRO: bisulfite pyrosequencing; NT: non-neoplastic thyroid; BTL: benign thyroid lesions; WDTC-GP: well-differentiated thyroid carcinomas of good prognosis; WDTC-PP: well-differentiated thyroid carcinomas of poor prognosis; ATC/PDTC: anaplastic carcinoma/poorly-differentiated thyroid carcinomas; TC-GP: thyroid carcinomas of good prognosis; TC-PP: thyroid carcinomas of poor prognosis; NS > 0.05; * *p* < 0.05; ** *p* < 0.01; *** *p* < 0.001.

The methylation levels of the CpGs were compared in thyroid samples from patients with poor prognosis (PDTC/ATC and relapsed WDTC), good prognosis (WDTC without relapse), and non-neoplastic tissues (NT and BTL). *PFKFB2* and *CXXC5* were hypomethylated in both tumor groups compared to NT and BTL. Moreover, *PFKFB2* methylation loss was more pronounced in poor prognosis cases. *ATPV6V0C* hypermethylation was observed in poor prognosis patients compared with good prognosis and non-neoplastic tissues (Figure 2A). *PFKFB2* hypomethylation and *ATPV6V0C* hypermethylation were also detected in poor-prognosis WDTC (WDTC-PP) compared with good-prognosis WDTC (WDTC-GP) cases (*p* = 0.003 and *p* = 0.027, respectively) (Figure 2B). However, considering exclusively the microarray-independent WDTCs samples (*N* = 79), only *PFKFB2* remained statistically significant (*p* = 0.031) (Figure S2).

### 2.3. Methylation Levels of PFKFB2 as Recurrence Predictor in WDTC

The predictive value of the CpGs methylation to discriminate WDTC-PP from WDTC-GP was tested verifying the area under the receiver operating characteristic curve (AUC) from our previous methylation microarray results [20], the TCGA methylation microarray database, and in samples from the current study assessed by bisulfite pyrosequencing (Figure S3). The *PFKFB2* methylation level was a promising recurrence predictor marker (methylation microarray analysis: AUC = 0.797; TCGA database: AUC = 0.667; current study: AUC = 0.698).

### 2.4. Lower Methylation Level of PFKFB2 is an Independent Marker of High Risk of Recurrence in WDTC

**Figure 3:**
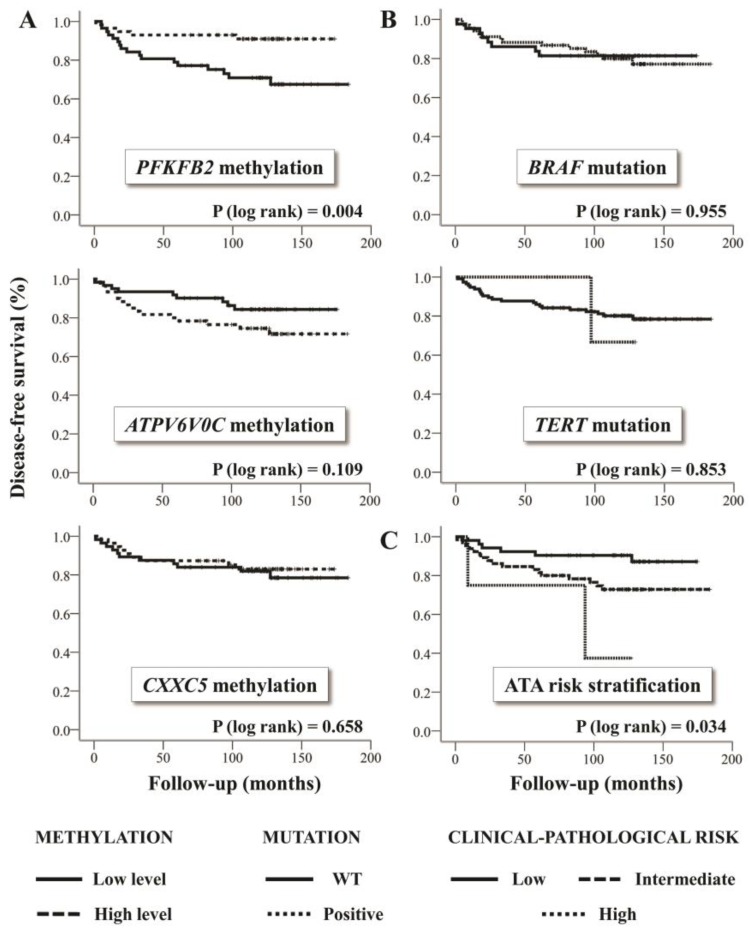
(**A**) Association of DNA methylation level (low and high) with disease-free survival. The survival curve demonstrates a shorter disease-free survival time in patients with lower methylation level of the evaluated CpG in *PFKFB2* promoter. (**B**) *BRAF* mutation (V600E) and *TERT* promoter mutation (C228T and C250T) in relation to disease-free survival. These alterations were not related to the relapse risk in the studied cohort. *BRAF* mutation evaluation (pyrosequencing) was performed only for papillary thyroid carcinomas (PTC) samples, and *TERT* promoter mutation assay (Sanger sequencing) was performed for all well-differentiated thyroid carcinomas (WDTC). (**C**) Disease-free survival according to the recurrence risk categories considering the clinical-pathological features (American Thyroid Association recurrence risk stratification method) [4]. Low methylation levels: below the median; high methylation levels: above the median; WT: wild type.

Shorter disease-free survival was observed in WDTC patients showing decreased methylation levels of *PFKFB2* (classified as below of the median) (Figure 3A). The *BRAF*V600E mutation was detected in 68 of 112 (60.7%) PTC samples and no increased risk of recurrence was verified (Figure 3B). High-quality results for *TERT* genotyping were obtained for 121 of 124 tumor samples. *TERT* promoter mutation was detected in two of three PDTC/ATC, and in four of 118 WDTC cases (3.4%) (three PTC and one FTC), showing no association with disease-free survival (Figure 3B).

Among the WDTC harboring *TERT* mutation, two PTC also presented *BRAF* mutation. In addition, hypomethylation of *PFKFB2* (*p* < 0.001) and *CXXC5* (*p* < 0.001) were associated with *BRAF* mutation (Figure S4). The multivariate analysis revealed *PFKFB2* hypomethylation as an independent factor of high risk of relapse (Hazard Ratio = 3.2; CI_95%_ = 1.1–9.5) (Table 1).

## 3. Discussion

We previously reported a DNA methylation signature composed of 21 CpGs which was capable of discriminating WDTC samples according to the clinical outcome (86% of accuracy). The reproducibility of this algorithm was corroborated using the TCGA-thyroid samples as an independent cohort of cases (82% accuracy) [20]. Here, three CpGs mapped in the gene promoters (*PFKFB2, ATP6V0C,* and *CXXC5*), included in this classifier, were selected to be evaluated by bisulfite pyrosequencing and to validate their potential to be used in the clinical practice.

The comparison between the DNA methylation values obtained by microarray and pyrosequencing analyses revealed high concordance (r > 0.75 for the three candidate markers tested). As expected, the same methylation pattern of *PFKFB2*, *CXXC5* (tumor hypomethylation in relation to NT and BTL), and *ATPV6V0C* (tumor hypermethylation in comparison with NT and BTL) described in the microarray analysis and in the TCGA cross-study validation [20] was confirmed. Furthermore, WDTC-PP patients presented significantly *ATP6V0C* increased and *PFKFB2* decreased CpGs methylation levels mapped in gene promoter. *ATP6V0C* encodes a component of vacuolar ATPase in a complex that regulates the acidification of eukaryotic intracellular organelles, playing a role in critical processes, zymogen activation, intracellular protein sorting, and endocytosis [24]. We integrated the methylation microarray and RNA sequencing data of 563 thyroid samples from TCGA (collected from UCSC Xena https://xenabrowser.net/datapages/—accessed in March 2019), and no significant association was found for *ATP6V0C* (Spearman correlation test *r* = 0.031; *p* = 0.459) (Figure S5). Nonetheless, an accurate prognostic marker could be a passenger alteration with no relevant biological function in a specific tumor type [25].

The CpG mapped in the *PFKFB2* promoter was demonstrated to be a promising prognostic marker based on the association with shorter disease-free survival and decreased DNA methylation levels. *PFKFB2* encodes a regulatory protein of the glycolytic pathway, which plays a role in the synthesis and degradation of fructose-2,6-bisphosphate [26]. *PFKFB2* increased expression levels were associated with shorter overall survival in ovarian cancer, showing a direct association with the long non-coding *LINC00092* [27]. These authors described that *PFKFB2* is an independent prognostic marker (*p* = 0.036, HR = 1.36). Functional in vitro and in vivo assays demonstrated induction of anoikis and loss of invasiveness by *PFKFB2* silencing [27]. *PFKFB2* was also reported as a potential thyroid cancer diagnostic marker, being down-expressed in malignant compared to benign thyroid tissues [28]. The integrative analysis between *PFKFB2* promoter methylation and gene expression from TCGA database (same parameter used to test *ATP6V0C*) revealed a significant positive correlation (r = 0.310; *p* < 0.001) (Figure S5). In addition, no association between *PFKFB2* expression levels with disease-free survival was detected using the TCGA database (log rank test *p* = 0.288). Gene expression control is a multifaced process, involving not only DNA methylation but a combination of several other epigenetic mechanisms, including histone modifications and non-coding RNA targeting [29]. In line with this, we previously suggested that *PFKFB2* is regulated by two miRNAs (hsa-miR-21 and hsa-miR-146b overexpression) that are putatively regulated by DNA hypomethylation [30]. These findings exemplify the complexity of the intercommunication among epigenetic mechanisms in gene expression regulation.

The *BRAF* V600E and *TERT* promoter mutations (C228T and C250T) have been extensively related to prognosis in thyroid cancer [6,7,8,11,12,13,31]. In our PTC cohort, 69 of the 113 cases (61% of frequency) presented *BRAF* mutation, a similar prevalence reported by other studies (50–70%) [32,33]. Overall, no difference in the relapse rate for patients harboring *BRAF* mutation was observed in our study.

*TERT* promoter mutations were detected in two of the three highly aggressive PDTC/ATC, in agreement with previous literature data [1,14]. However, this alteration was rare in our WDTC sample set, being detected in only 3.4% of the cases (four of the 118). A similar frequency was detected in WDTC-PP (one of 24 samples, 4.2%) and WDTC-GP (three of 94 WDTC-PP, 3.2%) cases. Therefore, the majority of patients showing recurrence were negative for *TERT* promoter mutations, and this marker failed to correctly predict the risk of recurrence in our cohort. The co-occurrence of *TERT* and *BRAF* mutations was observed in two PTC, and none of them relapsed.

Although our sample size was relatively small, the patients were carefully selected in a large period of follow-up (minimum of 5-years and median of 10.6 years). Of note, 65% (63 of 97) of the WDTC-GP patients were followed up for more than 10-years. Recurrence of WDTC has been described up to 43 years after the initial treatment [23]. However, the risk decreases in each subsequent year in the logarithmic trend, commonly occurring within the first five years [22].

Considering that *BRAF* mutation was detected in more than half of our PTC and most of the patients present indolent disease, other molecular mechanisms might be responsible for a more aggressive tumor phenotype. On the other hand, *TERT* promoter mutation is very uncommon in indolent WDTC and frequent in highly aggressive thyroid neoplasia. However, this alteration was not able to predict relapse in our WDTC cohort.

In conclusion, the DNA methylation analysis of *PFKFB2* promoter is a potential tool to estimate the risk of recurrence in WDTC patients, which can be easily performed by a low-cost technique compatible to the clinical practice as bisulfite pyrosequencing.

## 4. Materials and Methods

### 4.1. Patients

A cohort of 121 snap-frozen post-surgical WDTC samples was retrospectively included in this study, being 42 previously evaluated by global DNA methylation analysis (array-dependent) and 79 independent samples [20] (Table 2 and Table S1).

Additionally, three highly aggressive carcinomas (one PDTC and two ATC), 22 benign lesions, and 13 non-neoplastic thyroid tissues were included in this study. The patients were submitted to surgical procedures from 2001 to 2013 at A.C. Camargo Cancer Center, São Paulo, Brazil. The Institutional Human Research Ethics Committee of the Antonio Prudente Foundation at the A.C. Camargo Cancer Center approved the study (Protocol #2327-17, 21 February 2017). The study was conducted in accordance with the Helsinki Declaration.

Well-differentiated thyroid carcinoma patients classified as having “good-prognosis” (WDTC-GP) included those with no active disease (normal serum thyroglobulin measurement and imaging tests) during at least five years of follow-up (*N* = 96). “Poor-prognosis” patients (WDTC-PP) included cases with local recurrence (confirmed by imaging tests or histopathological analysis) or distant metastasis during the follow-up (*N* = 25).

Patients with incomplete clinical and pathological data, presenting the previous history of malignant tumors and relapsed after partial thyroidectomy or inadequate Thyroid-Stimulating Hormone (TSH) suppression, were excluded from the study.

### 4.2. Sample Processing, DNA Extraction, and Detection of BRAF and TERT Mutation

Samples were macrodissected, and DNA was isolated using conventional phenol-chloroform protocol and quantified by Qubit^®^ dsDNA BR Assay no Qubit^®^ 2.0 Fluorometer (Life Technologies, Carlsbad, CA, USA), as previously described [20].

*BRAF* V600E mutation was investigated exclusively in PTC samples by pyrosequencing using a threshold of 10% of altered alleles, as previously described [34]. The *TERT* promoter mutations, C228T and C250T (positions 124 and 146 base pairs upstream of the ATG site, respectively) were evaluated by direct Sanger sequencing after the amplification of the target regions (Forward primer: 5′CACCCGTCCTGCCCCTTCACCTT′3; Reverse primer: 5′GGCTTCCCACGTGCGCAGCAGGA′3), as previously described [35]. The amplification reaction was performed with HotStart Taq DNA polymerase (Qiagen, Valencia, CA, USA) and purified with ExoSAP-IT (USB Corporation, Cleveland, Ohio, USA). The sequencing reaction (reverse strand) was performed using ABI Prism BigDye Terminator Kit (Applied Biosystems, Foster City, CA, USA) in an ABI 3130xl DNA sequencer (Applied Biosystems, Foster City, CA, USA). The sequences were aligned using CLCBio Genomics Workbench Software (CLCbio’s, Aarhus, Denmark). Mutations were further confirmed by repeating the sequencing in both directions (forward and reverse).

### 4.3. Bisulfite Pyrosequencing for DNA Methylation Analysis

Three (cg02710090, cg05884711, and cg19628988) of 21 promoter CpGs differentially methylated probes, detected in WDTC-PP compared to WDTC-GP, were selected based on our previous study [20]. These three probes presented the highest AUC and were validated in the TCGA database analysis (Δβ between WDTC-PP and WDTC-GP >0.1 or <−0.1) (Table S2).

The DNA was denatured and treated with sodium bisulfite using the EZ DNA Methylation-Gold™ Kit (Zymo Research, Irvine, CA, USA), according to the manufacturer’s recommendations. The PCR followed by pyrosequencing was carried out as previously reported [34]. The primer sequences used were: *PFKFB2* (forward: 5′AGGTGGTGGTAGTGTAAGGA3′; reverse: 5′GAGAGGGTAATTTAGAGTATTTTGGGAG3′; sequencing 5′TTGAATTTTAAAGTA3′), *CXXC5* (forward 5′GGTAGGATTAGTTTAGGAGA3′; reverse 5′CTTCTAAAACACCACATAAAAAC3′; sequencing 5′TAGAGTGTATATTT3′), and *ATP6V0C* (forward 5′GGTTTTGAAGGTGTAGGTTTTG3′; reverse 5′CCACACCTATAAAATCCCAACC3′; sequencing 5′AGGTAGAGATAGGTG3′). Pyrosequencing reactions were assembled using the PSQ Vacuum Prep Tool (Qiagen) and sequenced at PSQ HS 96A Pyrosequencer (Qiagen) using Pyromark Gold Q96 (Qiagen).

### 4.4. Data Analysis

Statistical analysis was performed using SPSS (SPSS v. 21.0, Chicago, IL, USA) and GraphPad Prism 5.0 (GraphPad Software Inc., La Jolla, CA, USA). Microarray and bisulfite pyrosequencing methylation values were compared using Spearman’s correlation test. The sample groups were compared using Kruskal-Wallis (Dunn’s post-hoc) and Mann Whitney test. The performance of the methylation markers in discriminate WDTC-PP from WDTC-GP was verified by the AUC. Disease-free survival analysis was performed using the Kaplan-Meier estimator and log-rank test. Cox Proportional-Hazards Model was applied to perform the univariate and multivariate analyses (*p* < 0.10 in univariate analysis was fitted in the multivariate model). The null hypothesis was rejected with two-tailed *p* < 0.05.

## Figures and Tables

**Table 1:** Univariate and multivariate analyses of the comparison of clinical, pathological, and molecular characteristics of WDTC samples in relation to the risk of relapse.

Variable	Univariate Analysis		Multivariate Analysis	
	HR (CI_95%_)	*p*	HR (CI_95%_)	*p*
**Age**				
<55 years	1.0			
≥55 years	0.48 (0.11–2.05)	0.325		
**Gender**				
Female	1.0		1.0	
Male	3.63 (1.65–7.98)	**0.001**	1.89 (0.65–5.47)	0.242
**Tumor Size (cm)**				
≤1 cm	1.0			
>1 cm	1.69 (0.71–4.05)	0.239		
**Multicentricity**				
No	1.0		1.0	
Yes	2.10 (0.95–4.63)	0.066	1.53 (0.58–4.05)	0.396
**Histology**				
PTC	1.0			
FTC	1.08 (0.25–4.57)	0.919		
**PTC Variant**				
Classic	1.0			
Others	0.43 (0.13–1.43)	0.169		
**Extrathyroidal Extension**				
No	1.0		1.0	
Yes	1.96 (0.89–4.31)	0.093	1.53 (0.41–5.61)	0.525
**Lymph node Metastasis**				
No (cN0, pN0)	1.0		1.0	
Yes (pN1)	4.19 (1.85–9.51)	**<0.001**	5.77 (0.64–52)	0.118
Risk stratification *				
Low	1.0		1.0	
Intermediate	2.49 (0.98–6.33)	0.055	0.35 (0.03–4.54)	0.419
High	6.08 (1.22–30.35)	**0.028**	0.35 (0.01–12.36)	0.563
***BRAF*V600E #**				
No	1.0		1.0	
Yes	0.98 (0.42–2.26)	0.955	0.74 (0.27–2.02)	0.560
***TERT* C228T/C250T**				
No	1.0			
Yes	1.21 (0.16–8.95)	0.854		
***CXXC5 Methylation***				
Below Median	1.22 (0.51–2.94)	0.658		
Above Median	1.0			
***ATP6V0C Methylation***				
Below Median	1.0			
Above Median	1.93 (0.85–4.36)	0.116		
***PFKFB2 Methylation***				
Below Median	3.85 (1.42–10.44)	**0.008**	3.17 (1.06–9.46)	**0.038**
Above Median	1.0		1.0	

HR: hazard ratio; *p*: obtained from Cox regression model. CI_95%_: 95% confidence interval; PTC: papillary thyroid carcinoma; FTC: follicular thyroid carcinoma; cN0: no clinical evidence of lymph nodes involvement; pN0: no pathologically evidence of lymph nodes involvement; pN1: pathological confirmation of lymph nodes involvement; * American Thyroid Association recurrence risk stratification [4]; # variable entered in the multivariate model, since an association with *PFKFB2* methylation was detected; bold: significant *p*-value.

**Table 2:** Clinical and pathological features of well-differentiated thyroid carcinomas (WDTC) patients enrolled in the study.

Characteristics	Microarray Dependent		Microarray Independent	
	*N* = 42	%	*N* = 79	%
**Age**				
Median (interquartile range)	40.4 (31.4–49.9)		44.2 (34.5–51.0)	
<55 years	35	83.3%	67	84.8%
≥55 years	7	16.7%	12	15.2%
**Gender**				
Female	36	85.7%	54	68.4%
Male	6	14.3%	25	31.6%
**Histology**				
PTC classic variant	28	66.7%	56	70.9%
PTC follicular variant	4	9.5%	15	19.0%
PTC rare variant	4	9.5%	5	6.3%
FTC	6	14.3%	3	3.8%
**Tumor dimension (cm)**				
Median (interquartile range)	1.3 (0.9–1.9)		1.4 (1.0–2.2)	
≤1 cm	15	35.7%	31	39.2%
>1 cm	27	64.3%	48	60.8%
**Multicentricity**				
No	32	76.2%	42	53.2%
Yes	10	23.8%	37	46.8%
**Extrathyroidal extension**				
No	27	64.3%	48	60.8%
Yes	15	35.7%	31	39.2%
**Lymph node metastasis**				
No (cN0, pN0)	26	61.9%	53	67.1%
Yes (pN1)	16	38.1%	26	32.9%
Risk stratification *				
Low	16	38.1%	36	45.6%
Intermediate	23	54.8%	42	53.2%
High	3	7.1%	1	1.3%
**Clinical evolution**				
Free of disease	35	83.3%	61	77.2%
Relapsed	7	16.7%	18	22.8%

PTC: papillary thyroid carcinoma; FTC: follicular thyroid carcinoma; cN0: no clinical evidence of lymph node involvement; pN0: no pathological evidence of lymph node involvement; pN1: pathological confirmation of lymph node involvement; * American Thyroid Association recurrence risk stratification [4].

## Supplementary Materials

Supplementary materials can be found at https://www.mdpi.com/1422-0067/20/6/1334/s1.

## Abbreviations

ATC	anaplastic thyroid carcinoma
AUC	area under the curve
BTL	benign thyroid lesions
CpG	5-cytosine-phosphate-guanine-3
FTC	follicular thyroid carcinoma
NT	non-neoplastic adjacent tissues
PDTC	poorly-differentiated thyroid carcinoma
PTC	papillary thyroid carcinoma
TCGA	The Cancer Genome Atlas
TSH	Thyroid-Stimulating Hormone
WDTC	well-differentiated thyroid carcinoma
WDTC-GP	good-prognosis well-differentiated thyroid carcinoma
WDTC-PP	poor-prognosis well-differentiated thyroid carcinoma
