# Supplementary material for: PFKFB2 Promoter Hypomethylation as Recurrence Predictive Marker in Well-Differentiated Thyroid Carcinomas
Source: Int J Mol Sci. 2019 Mar 16;20(6):1334. doi: 10.3390/ijms20061334 (PMC6471408; doi:10.3390/ijms20061334)
Supplement: Supplementary file 1 [file ijms-20-01334-s001.zip › ijms-457776-supplementary.pdf]

International Journal of *Molecular Sciences*

MDPI

Supplementary Materials

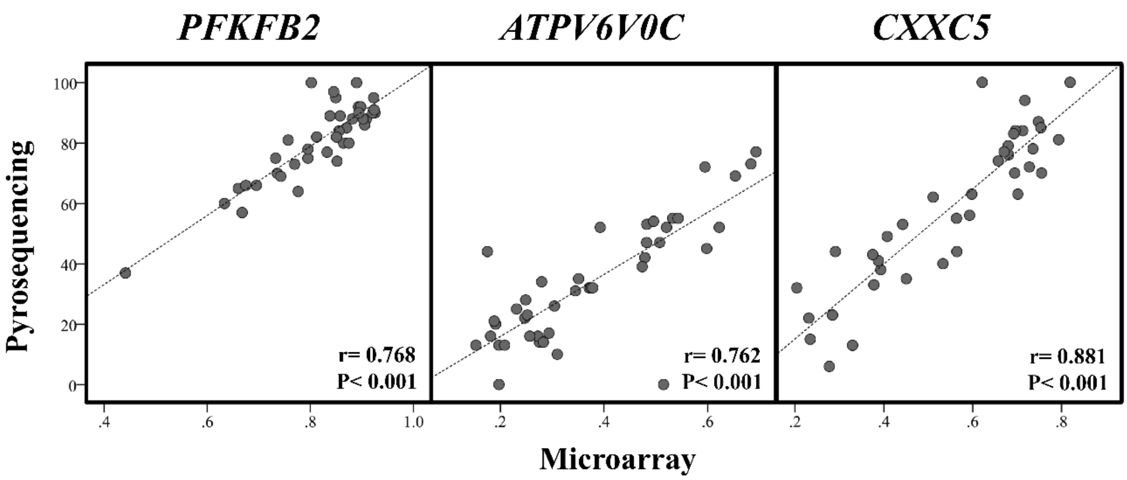

Figure 1. Correlation between values obtained by methylation microarray and bisulfite pyrosequencing assays. Scatterplots showing high values of positive correlation for *PFKFB2, ATPV6V0C* and *CXXC5*. r= correlation coefficient, P= p-value from Spearman's correlation test.

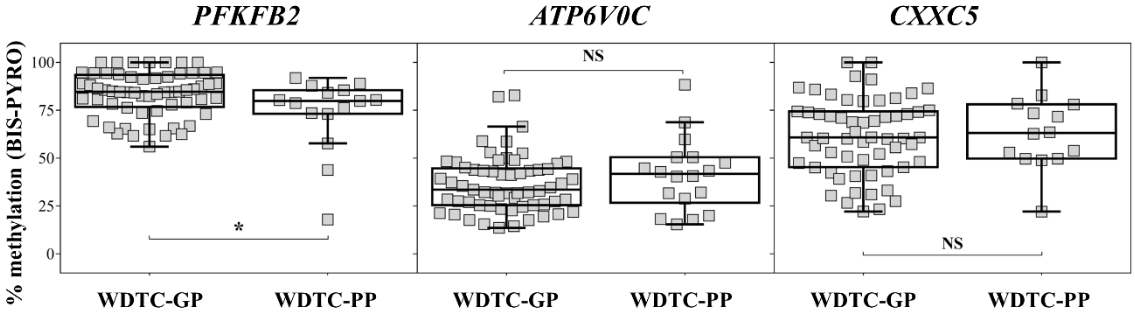

Figure 2. Bisulfite pyrosequencing quantification of CpG allele methylation mapped in *PFKFB2, CXXC5* and *ATPV6V0* in well-differentiated thyroid carcinomas (microarray-independent cases). The CpGs methylation levels in WDTC-PP patients were compared with WDTC-GP. BIS-PYRO: bisulfite pyrosequencing; NS> 0.05; *P< 0.05 (Mann-Whitney test); WDTC-GP: well-differentiated thyroid carcinomas of good prognosis; WDTC-PP: well-differentiated thyroid carcinomas of poor prognosis.

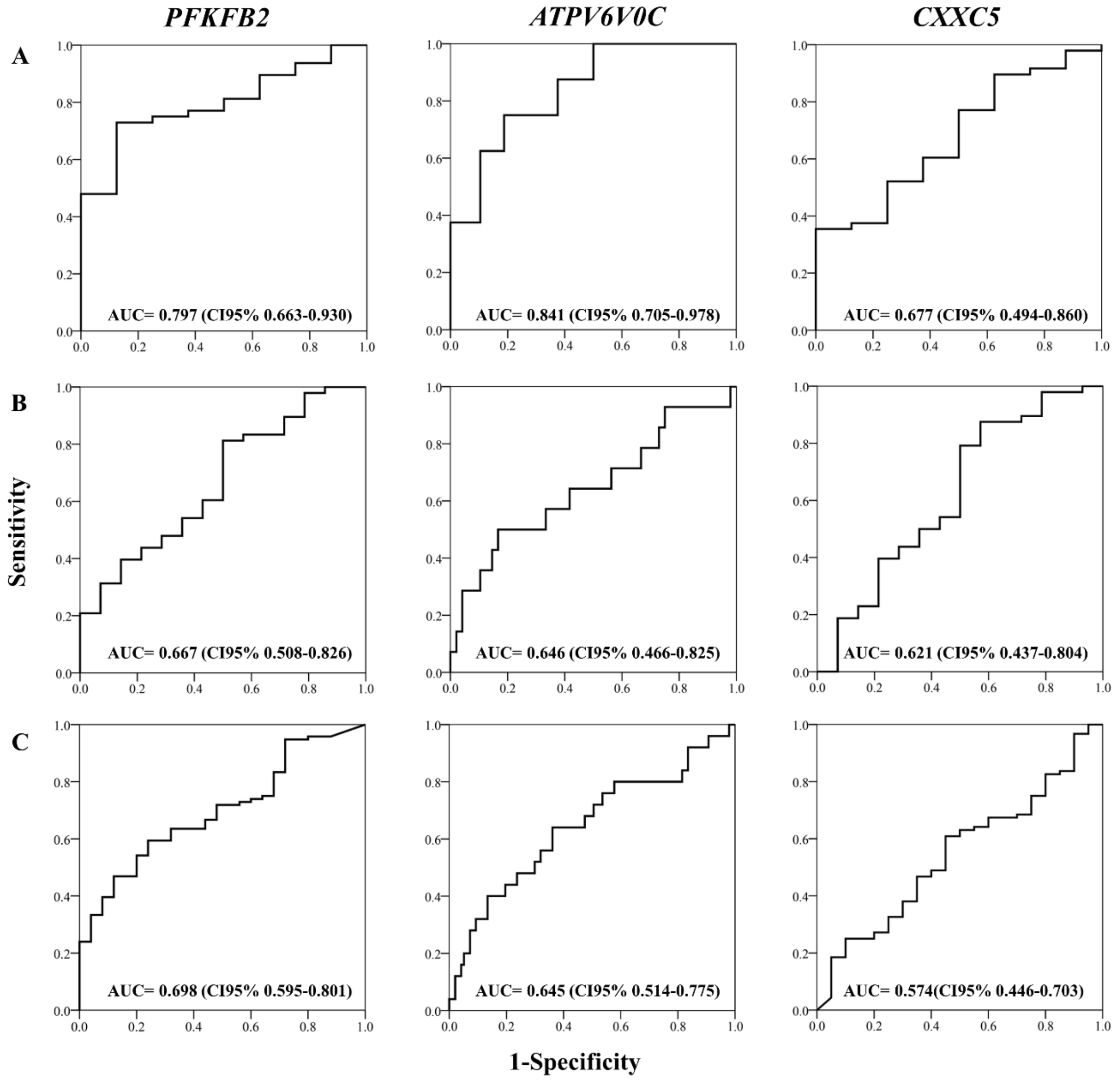

Figure 3. (**A**). Area under the ROC curve of the epigenetic candidate markers to identify aggressive subtypes of WDTC samples previously evaluated by methylation microarray (BISARRO DOS REIS et al., 2017); (**B**) TCGA cohort of PTC and (**C**) cases evaluated by bisulfite pyrosequencing. CI $_{95\%}$: 95% confidence interval; AUC: area under the receiver operating characteristic (ROC) curve.

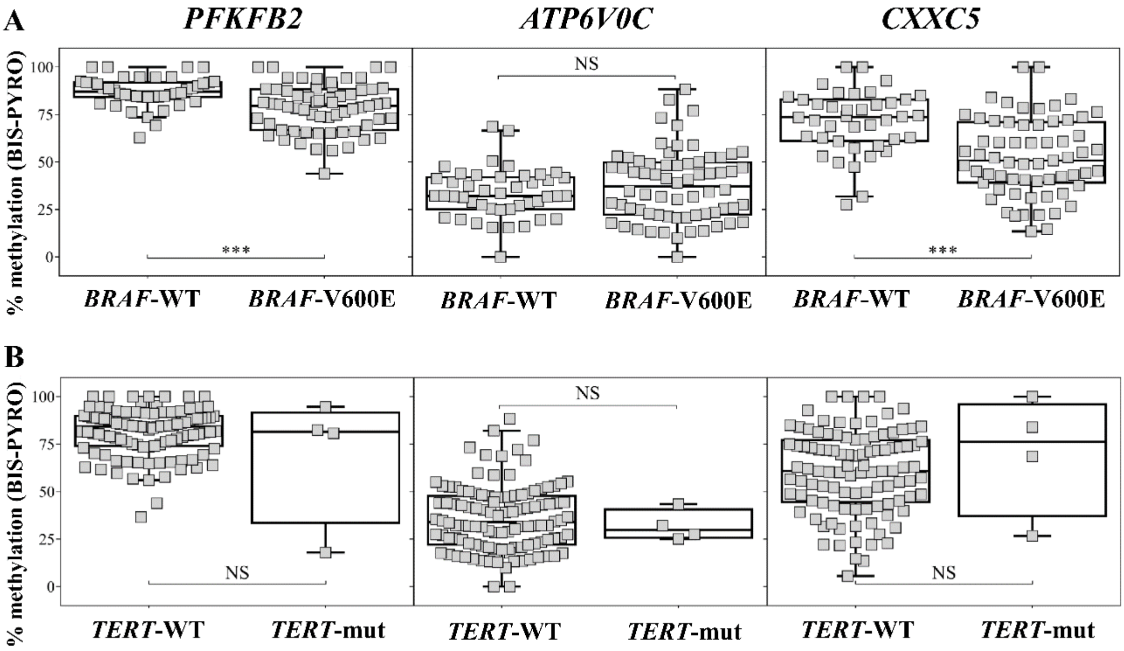

Figure 4. DNA methylation in *PFKFB2, ATP6B0C* and *CXXC5* according to *BRAF (*V600E) and *TERT* (C228T and C250T) mutation status. BIS-PYRO: bisulfite pyrosequencing; NS> 0.05; ***P< 0.001 (Mann-Whitney test).

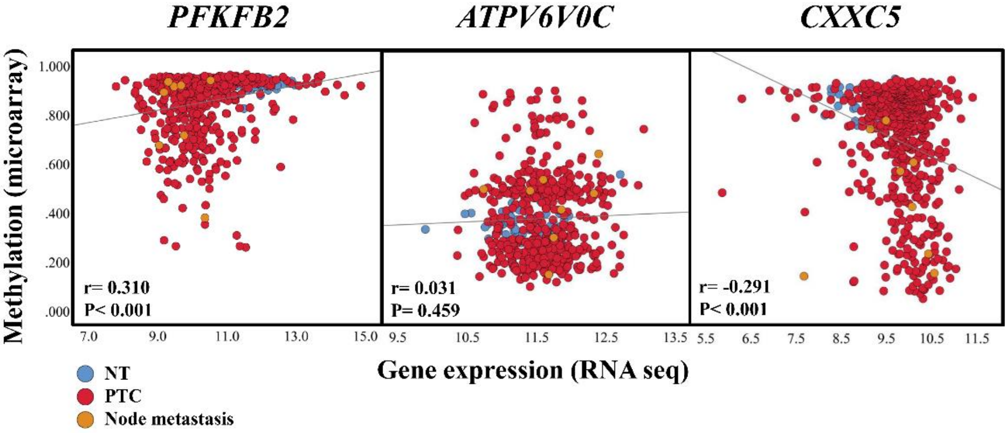

Figure 5. Correlation between CpG methylation and gene expression levels of the studied genes in the thyroid cohort from TCGA database. A positive correlation was observed between DNA methylation (probe cg02710090) and *PFKFB2* expression and negative correlation between DNA methylation (probe cg19628988) and *CXXC5*. No correlation was noted to *ATP6V0C* (methylation probe cg05884711). r= correlation coefficient, P= p-value from Spearman's correlation test. NT: non-neoplastic thyroid; PTC: papillary thyroid carcinoma.
